# Effect of semi-recumbent vibration exercise on muscle outcomes in older adults: a pilot randomized controlled clinical trial

**DOI:** 10.1186/s12877-022-03052-0

**Published:** 2022-04-18

**Authors:** Murad H. Taani, Neil Binkley, Ronald Gangnon, Diane Krueger, Bjoern Buehring

**Affiliations:** 1 University of Wisocnsin Milwaukee, Wiscosin State, Milwaukee, USA; 2 University of Wisocnsin Madison, Wiscosin State, Madison, USA

**Keywords:** Exercise, Muscle function, Jump power, Residential care apartment complexes

## Abstract

**Background:**

Many older adults with physical limitations living in residential care apartments are unable to exercise in a standing position and are at risk for declining in muscle function leading to falls and injury. Novel approaches to achieve exercise benefits are needed. The purpose of this study was to test the effect of semi-recumbent vibration exercise on muscle outcomes in older adults living in residential care apartment complexes (RCACs).

**Methods:**

A randomized, crossover design was used to examine the effect of semi-recumbent vibration exercise on muscle function and mass among 32 RCAC residents (mean age 87.5 years) with physical limitations. Participants received a randomized sequence of two study conditions: sham or vibration for 8 weeks each separated by a 4-week washout. Before and after the 8 weeks of vibration treatment and sham treatment, muscle mechanography was used to assess muscle function including jump power, weight-corrected jump power, and jump height. Short physical performance battery (SPPB) and handgrip strength were also used to measure muscle function. Bioelectrical impedance spectroscopy was used to estimate skeletal muscle mass. The effect of the vibration treatment on muscle outcomes was analyzed through mixed effects linear regression models.

**Results:**

Vibration exercise leads to better jump height (*p* < .05) compared to sham exercise but also poorer chair rise performance (*p* = 0.012). Other muscle functions tests and muscle mass parameters showed non-significant changes.

**Conclusion:**

This small pilot study showed no conclusive results on the effect of semi-recumbent vibration exercise on muscle function and mass in older adults living in RCAC. However, the promising signals of improved jump performance could be used to power larger studies of longer duration with various vibration doses to determine the benefit of vibration exercise in this physically impaired, high-risk population with few exercise capabilities.

**Trial registration:**

The study is registered at clinicaltrials.gov (NCT02533063; date of first registration 26/08/2015).

## Background

Ageing is accompanied by body composition changes that include reduction in muscle mass and loss of muscle strength or function (i.e., sarcopenia) [1]. These changes have significant implications for physical function among older adults such as reductions in power, gait, and balance and increased risk for falls. These reductions are associated with impaired physical function, dependency, and reduced quality of life [1, 2]. As sarcopenia contributes to adverse outcomes, exercise programs to mitigate loss of muscle mass, strength, and function are desirable [3]. Resistance exercise has promise in improving physical function and muscle outcomes in older adults [2, 3]. However, many older adults do not or cannot routinely exercise; moreover even following exercise prescription adherence is a major challenge [4, 5]. As such, novel approaches providing exercise benefits to older adults have potential to substantially enhance physical function and quality of life for many older adults.

Vibration training has emerged as an attractive exercise intervention that is time efficient, effective and safe, particularly among frail and very old adults [6]. Vibration exercise activates skeletal muscle via linear accelerations transmitted by the vibration device into the musculoskeletal system. These stimuli lead to the muscle contraction by triggering the activity of α-motor neurons. Based on this mechanism, vibration exercise has been reported to improve muscle outcomes [7]. Importantly, vibration exercise requires less time than other exercise regimens and can be performed by older adults with comorbidities, e.g., cardiac or respiratory disease, that limits the ability to perform conventional exercise. Vibration training modalities differ; most vibration exercise systems require individuals to stand on a platform. Exercising in a standing position requires greater concentration and musculoskeletal coordination than sitting and may be too difficult for some individuals with physical function impairments or other co-morbidities. To obviate this important limitation, a novel vibration system was developed by VibeTech™ (Sheboygan, WI) providing semi-recumbent vibration exercise.

Most prior studies examined the effect of whole-body vibration (WBV) exercise and have involved community dwelling older adults [6, 8]. Moreover, older adults living in residential care apartment complexes (RCACs)—where they receive a variety of services e.g., personal assistance, and nursing services based on their specific needs are at greater risk for declining of muscle function thereby necessitating moving to more restrictive living environments such as nursing homes [9]. The purpose of this study was to examine the effect of semi-recumbent vibration exercise on muscle function and mass in older adults living in an RCAC. We hypothesized that muscle function and mass would improve after the semi-recumbent vibration exercise.

## Methods

### Design, Setting, and Sample

**Fig. 1:**
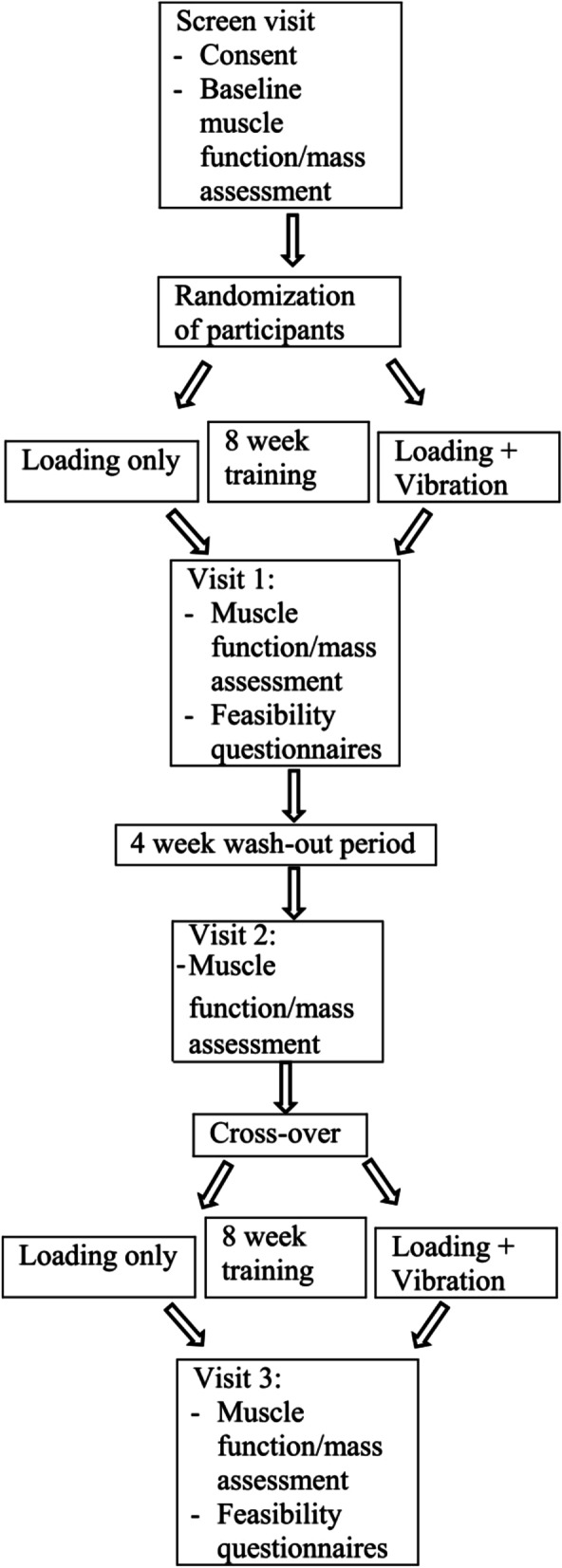
Study flowchart

A randomized, crossover design was used in which participants received a randomized sequence of two study conditions: sham and vibration exercise for 8 weeks each with a 4-week washout period. The details of the study design including methodology, feasibility, and safety are described elsewhere [10]. Figure 1 shows study flowchart. Briefly, 32 older adults with decreased physical function defined by decreased short physical performance battery (SPPB) score were recruited from one RCAC in the Midwestern United States between November 2015 and February 2017. RCACs are independent apartment complexes that provide not more than 28 h per week of a variety of services to those residing in the complex based on the individual's specific needs. Those services include supportive services (e.g., activities related to general housekeeping and recreational activities), personal assistance (e.g., services related to activities of daily living such as dressing, eating, bathing and grooming), and nursing services (e.g., health monitoring and medication management) [11]. Inclusion criteria were: English-speaking, age ≥ 70 years, capable of providing informed consent, ability to stand independently, free of major acute illness, a short SPPB score ≤ 9 or ≥ 2 in any of the three sections (balance, gait speed, or chair rise), willing to train for 10 min, 3 times per week for two 8-week periods. Exclusion criteria were not able to stand without assistance (use of cane or walker was allowed), history of injury or surgery in the past six months that limited mobility or ability to perform muscle and physical function tests, and major illness that might cause missed training sessions or visits. The study was approved by the institutional review board of the University of Wisconsin Madison (IRB#: 2015–0480) and written consent was obtained from the participants.

### Measurement

Data on age, sex, history of falls in the last year were obtained at baseline by an interviewer-administered questionnaire. Height, weight, and Body Mass Index (BMI) were also measured.

### Muscle function

Muscle mechanography was used to assess jump power (Watt), relative (weight corrected) jump power (Watt/kg), and jump height (meter) [12, 13]. A Leonardo force plate (Novotec Medical, Pforzheim, Germany) was used to perform the measurements. Each participant followed a standardized procedure [12] to perform three two-leg maximal countermovement jumps on the force plate deemed valid by the software. Participants were asked to jump as high as possible using both legs, attempting to touch the ceiling with their head. The jump with greatest height was used and maximal relative power of the jump was calculated and used for analyses.

The SPPB test consists of gait speed as determined by a four-meter walk, timed repeated chair rise, and standing balance tests [14]. Gait speed was measured by instructing participants to walk four meters at their normal pace; they are timed with a stopwatch. This test was repeated twice and the faster of the two walks used for analyses. The timed repeated chair rise has participants stand up from a chair five times without the use of their arms after demonstrating the ability to rise once without using their arms. Participants were seated on a firm seat at knee level with their arms crossed over the chest and are asked to stand up from and sit back down five times as quickly as possible. Time to complete five stands was measured. Standing balance was assessed by having the participants stand in three positions of increasing difficulty for 10 s each. This initially consists of the feet being placed side by side, subsequently the heel of one foot is placed alongside of the big toe of the other foot and finally a tandem position is utilized with one foot directly in front of the other. Each component has a possible score of 0 to 4 and the total score ranges from 0 to 12. The greater the score the better the physical performance.

Hand grip strength was measured using a hydraulic JAMAR handgrip dynamometer [15]. Participants performed three attempts using their non-dominant hand, resting 10 to 20 s between attempts, while sitting in an upward position with the arm in a 90-degree angle position. The highest score was used for analyses.

### Muscle mass

A tetrapolar bioelectrical impedance spectroscopy (BIS) (ImpediMed® SFB7) device was used to estimate skeletal muscle mass. The details of BIS have previously been described [16, 17]. BIS measures the dynamic resistance across a spectrum of electrical frequencies through the body to distinguish between intracellular fluid (ICF) and extracellular fluid (ECF) and thereby is able to measure muscle mass (ICF), not simply fat free mass (FFM; mostly ICF and ECF). Participants were asked to empty their bladder and remove all jewelry, then positioned in a supine position for a minimum of 10 min prior to measurement. Adequate separation of their legs was obtained to allow for accurate measurement. Measurements were obtained by placing four electrocardiogram-like electrodes on the skin of the participant’s hand and feet. Measurements were taken twice, and the mean scores were used for analyses.

### Semi-Recumbent Vibration Exercise and Study Procedure

Both the semi-recumbent vibration exercise and training protocol are detailed elsewhere [10]. Briefly, the VibeTech™ device is designed to allow individuals to receive vibration training to their legs while seated and performing leg presses against the device footplate. The footplate is force-driven with a robotic loading system that supplies between 5 and 100 lbs of force to the legs depending on the individual’s ability level. This force was increased as tolerated by the participant. The vibration frequency for this study was set at 30 Hz because previous reports suggested the most pronounced effect of vibration at this setting [18].

Participants completed the baseline assessment which included measurements of muscle function and mass following screening and before receiving any treatment. Participants were then randomly assigned using a 1:1 allocation ratio to initially receive vibration treatment or sham treatment by the study coordinator. Participants were informed that they will receive two different types of exercise, and we avoided the term ‘sham’ when communicating with the participants to minimize a placebo/nocebo effect. The data collector was blinded to the randomization. During the vibration treatment, participants were trained with leg loading and vibration for 10 min 3 times a week. During the sham treatment, participants were only seated in the vibration device and the device’s knee support was placed on their upper leg above the knee for 10 min 3 times a week without the vibration being administered. Before each training session, participants were asked to push as hard as possible on a regular weight scale that was placed on the footplate of the vibration device. The applied load was individualized according to the weight measured on the scale which was used as the load for the vibration treatment session. The vibration intensity level was also assessed and adjusted every 2 weeks. It was initially set to 0.2 g (Level 1) and then increased to level 2 (0.4 g), level 3 (0.6 g), and level 4 (0.8 g). At the end of the 8 weeks, muscle function and mass were measured by a data collector blinded to training assignment and participants received a 4-week washout period, i.e., no vibration or sham training. The length of the washout period was based on a literature review on how long effects of vibration training last on average. At the end of the washout period the same measurements were conducted again and participants crossed-over to the opposite treatment to receive the other treatment 3 times per week for 8 weeks as noted above. At the end of the 8 weeks, the same measurements of muscle function and mass were conducted.

### Data Analysis

Descriptive statistics was used to present the demographic characteristics of the participants and study variables. The data were analyzed using a mixed effects linear regression model with the covariance structure proposed in Kenward and Roger [19] for crossover trials with baseline measurements. The mixed effects model properly accounts for missing data under a missing-at-random assumption. Analyses were performed using Proc Mixed in SAS 9.4 (Cary, NC). Sample size estimation was conducted based on the primary outcome variable of relative jump power. To detect a 10% difference in maximum jump power between control and intervention groups, 26 subjects are needed per group (alpha = 0.05; 80% power; 2-sided) assuming a standard deviation of 2.6 and control group mean of 21 W/kg. These values are based on data previously collected by our group involving 81 older adults. Accounting for up to 20% subject withdrawal brings the required enrollment to 32 subjects per group (64 total). Using a cross-over design, the number of needed participants was 32 participants.

## Results

### Participant characteristics

**Fig. 2:**
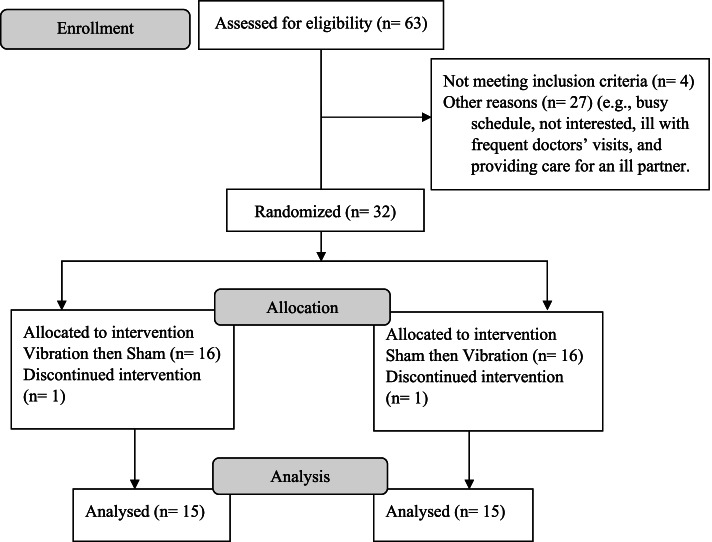
CONSORT diagram of progress through the enrolment, intervention allocation, follow-up, and data analysis of this study

Data of 30 participants were analyzed (Fig. 2). The study sample was comprised of older adults (87.5 ± 6.0 years.; range = 74.9, 99.0 years.; 21 women and 9 men). Participants’ baseline characteristics were as follows: height (157.0 ± 7.7 cm), weight (67.6 ± 11.6 kg), and BMI (27.5 ± 5.1 kg/m2). All other participants characteristics are depicted in Table 1.

**Table 1:** Demographics and baseline characteristics (*n* = 30)

	**Frequency (%)**
Female	21 (70%)
Falls Yes No	12 (40%) 18 (60%)
	**Mean (SD)**
Age	87.500 (6.000)
BMI	27.534 (5.124)
Jump Power (W)	0.619 (0.570)
Relative Jump Power (W/kg)	9.290 (8.190)
Jump Height (m)	0.080 (0.064)
Balance Score	2.670 (1.248)
Gait Speed (m/s)	0.671 (0.171)
Chair Rise (s)	17.260 (5.961)
SPPB	6.910 (2.718)
Grip Strength (kg)	14.215 (5.964)
TBW (L)	29.735 (7.144)
ECF (L)	14.440 (3.737)
ICF (L)	16.240 (3.599)
FFM (KG)	41.375 (9.760)

Falls in the past one year; *W* Watt, Jump Height measured per meter; Balance score range score 0–4; Gait Speed measure as meter/second; Repeated chair rise time measured per second; Total SPPB range score 0–12; *TBW (L)* total body water measure per liter, *ECF (L)* extracellular fluid measure per liter, *ICF (L)*: intracellular fluid measured per liter, *FFM* fat free mass measured per kg

### Muscle function

Jump height after vibration exercise across the two study periods was 2.5 cm (95% CI 1–50 cm, *p* = 0.044) higher compared to sham exercise, while chair rise time was 2.6 s longer (95% CI 0.7–4.5 s, *p* = 0.012). Jump power and relative jump power were numerically higher after receiving vibration exercise compared to sham, although not statistically significant. Balance and gait speed scores were also numerically higher whereas SPPB and grip strength were numerically lower, without reaching significance. See Table 2 for details.

**Table 2:** Muscle function and mass outcomes

	Period 1			Period 2			Vibration vs Sham			
	Baseline	Sham	Vibe	Baseline	Sham	Vibe	Estimate	95% CI		*p*-value
**Muscle Function**										
Jump Power (W)	0.556	0.576	0.572	0.682	0.573	0.620	0.021	-0.035	0.077	0.41
Relative Jump Power (W/kg)	8.49	8.66	8.83	10.09	8.62	9.30	0.42	-0.45	1.30	0.29
Jump Height (m)	0.063	0.063	0.088	0.078	0.055	0.080	**0.025**	**0.001**	**0.050**	**0.044**
Balance Score	2.42	2.62	2.54	3.10	2.48	2.67	0.05	-0.29	0.39	0.77
Gait Speed (m/s)	0.652	0.701	0.674	0.701	0.679	0.710	0.001	-0.070	0.073	0.97
Chair Rise (s)	17.28	13.86	17.30	15.14	14.70	16.40	**2.59**	**0.66**	**4.53**	**0.012**
SPPB	6.35	6.89	6.68	7.47	7.27	7.09	-0.20	-0.80	0.40	0.50
Grip Strength (kg)	14.09	14.26	13.18	13.53	15.21	13.86	-1.21	-2.59	0.16	0.081
**Muscle Mass**										
TBW (L)	29.71	29.36	29.80	29.76	32.68	31.73	-0.13	-2.64	2.38	0.91
ECF (L)	15.13	14.95	14.89	13.75	15.38	14.55	-0.41	-1.63	0.82	0.49
ICF (L)	16.84	16.95	17.13	15.64	17.04	16.44	-0.18	-2.11	1.75	0.84
FFM (kg)	39.95	39.57	39.99	40.80	44.76	43.37	-0.32	-3.85	3.22	0.85

*W* Watt, Jump Height measured per meter; Balance score range score 0–4; Gait Speed measure as meter/second; Repeated chair rise time measured per second; Total SPPB range score 0–12, *TUG* Timed Up and Go test, *TBW (L) * total body water measure per liter, *ECF (L)* extracellular fluid measure per liter, *ICF (L) *intracellular fluid measured per liter, *FFM* fat free mass measured per kg

### Muscle mass

There were no differences in muscle mass  parameters (i.e., TBW, ECF, ICF, FFM) between the vibration and sham treatment. See Table 2 for details.

## Discussion

To our knowledge, this is the first study to examine the effect of semi-recumbent vibration exercise on muscle function and mass among older adults living in RCACs. This study demonstrated improvement in jump height and trends toward improvement in jump power, relative jump power, balance, and gait speed among the study participants after receiving vibration exercise. However, the results also showed significantly longer chair rise time and numerically lower SPPB and grip strength scores, but without reaching significance, after receiving vibration exercise. Although clear inconsistencies between muscle function tests have to be noted, these results suggest that semi-recumbent vibration exercise may have the potential to improve muscle function, including jump height and power, balance and gait speed in older adults with pre-existing physical function limitations.

Our findings and results from other studies using whole-body and local vibration exercise [20, 21] suggest that this novel semi-recumbent vibration exercise might have some benefit for the at-risk, very old older adults with physical function limitations. This population might not be able to exercise in a standing position because standing requires greater concentration and musculoskeletal coordination than sitting. The semi-recumbent exercise may obviate these barriers and serve as an effective exercise modality. Thus, a larger trial powered on the maximal jump height differences in this pilot study could potentially prove the positive effects suggested in this study.

An unexpected finding of this study were the improvements in chair rise after the sham treatment compared to the vibration intervention. We can only speculate that this finding might be an effect from the small sample size where larger individual changes influence the overall average more than in larger samples. This study population of older adults is frequently experiencing some level of health impairment which likely impacts their muscle function test performance. Such variability resulting from intercurrent illness should be taken into account when designing similar larger studies of functionally impaired older adults.

It is also noteworthy to mention that we found this exercise safe and feasible with good adherence in our previous report [10]. Moreover, while all participants were able to complete the test battery (questionnaires, muscle function, and muscle mass measurements), the tests in a such population need to focus on a small number of parameters. Too many tests can create a burden on the participants, which can cause test fatigue and inconsistent test results.

Our study did not demonstrate an improvement in muscle mass. This finding is consistent with previous studies reporting that muscle mass might not increase after receiving whole-body or local vibration exercise in older adults [20–22]. Research reported that vibration exercise might not provide sufficient stimulus to reverse skeletal muscle hypertrophy in older adults and revealed no benefit of vibration exercise on muscle mass among frail older adults, nursing home residents, or those with physical limitations, such as our sample [20, 21]. The lack of muscle mass improvement could be explained, in part, by an insufficient dose of vibration exercise or the vibration treatment period being too short. Many older adults are unable to tolerate higher doses of vibration training and small doses are inadequate to improve muscle mass [22]. Further, research indicated that vibration exercise in a standing position has greater benefit and may facilitate muscle response to the vibration [23] while vibration exercise in half-squatting and sitting positions may reduce the effect of vibration and make it insufficient to increase muscle mass in the participants [21, 22, 24].

Our study has limitations. Given the nature of a pilot trial, our study included a small number of participants over a short period of time, which might have precluded the ability to detect significant effects on muscle function and mass and generalize the study findings. Using a large sample and a longer duration of vibration treatment could potentially show improvement. Additionally, these results may not be generalizable to some older adults including those that are healthier than our study population or those with more severe physical limitations. Further, while we informed the participants that they will receive two different types of exercise and we avoided the term ‘sham’ when communicating with the participants, some participants may know which condition might have better effect (i.e., vibration training), leading to psychological influence on the outcome measurements (i.e. placebo / nocebo effect). The study was performed at a single RCAC so there might have been a bias in selection of the study subjects. Another limitation is that the BIS method may underestimate muscle mass due to the common problem of dehydration among older adults which was not assessed in our study. Thus, further studies are needed to overcome these study limitations.

## Conclusions

In conclusion, our study reveals that the semi-recumbent vibration exercise has some promising signals in improving jump performance among older adults with physical limitations, including very old individuals. Future larger studies of longer duration and potentially higher doses of vibration training building on the findings of the current study are warranted to explore the potential of targeting decreased muscle function and mass to benefit older adults living in RCACs.

## Data Availability

The datasets generated during and/or analyzed during the current study are not publicly available as participants did not provide consent for their data to be shared, but further details about the data and ethical conditions for access are available from the corresponding author on reasonable request.

## Abbreviations

RCACs: Residential care apartment complexes
WI: Wisconsin
WBV: Whole-body vibration
SPPB: Short physical performance battery
BMI: Body mass index
BIS: Bioelectrical impedance spectroscopy
ICF: Intracellular fluid
ECF: Extracellular fluid
FFM: Fat free mass
SD: Standard deviation
CI: Confidence interval
